# Hierarchical Sparse Learning with Spectral-Spatial Information for Hyperspectral Imagery Denoising

**DOI:** 10.3390/s16101718

**Published:** 2016-10-17

**Authors:** Shuai Liu, Licheng Jiao, Shuyuan Yang

**Affiliations:** 1 Key Laboratory of Intelligent Perception and Image Understanding of Ministry of Education, Xidian University, Xi’an 710071, China; lchjiao@mail.xidian.edu.cn (L.J.); syyang@xidian.edu.cn (S.Y.); 2 Joint International Research Laboratory of Intelligent Perception and Computation, Xidian University, Xi’an 710071, China

**Keywords:** hierarchical sparse learning, denoising, spectral-spatial information, hyperspectral images

## Abstract

During the acquisition process hyperspectral images (HSI) are inevitably corrupted by various noises, which greatly influence their visual impression and subsequent applications. In this paper, a novel Bayesian approach integrating hierarchical sparse learning and spectral-spatial information is proposed for HSI denoising. Based on the structure correlations, spectral bands with similar and continuous features are segmented into the same band-subset. To exploit local similarity, each subset is then divided into overlapping cubic patches. All patches can be regarded as consisting of clean image component, Gaussian noise component and sparse noise component. The first term is depicted by a linear combination of dictionary elements, where Gaussian process with Gamma distribution is applied to impose spatial consistency on dictionary. The last two terms are utilized to fully depict the noise characteristics. Furthermore, the sparseness of the model is adaptively manifested through Beta-Bernoulli process. Calculated by Gibbs sampler, the proposed model can directly predict the noise and dictionary without priori information of the degraded HSI. The experimental results on both synthetic and real HSI demonstrate that the proposed approach can better suppress the existing various noises and preserve the structure/spectral-spatial information than the compared state-of-art approaches.

## 1. Introduction

As three-dimensional (3D) data integrating both spectral and spatial information concurrently, hyperspectral imagery (HSI) can provide more reliable and accurate information about observed objects. It has numerous applications in remote sensing, diagnostic medicine and mineralogy, etc., with encouraging results [1,2,3]. HSI can be considered as a set of gray-scale images over wavelength, whose entries are the spectral responses. Due to the limited light, imaging system, photon effects, and calibration error, hyperspectral images are inevitably contaminated by annoying noises with different statistical properties [4,5]. The existing noises greatly reduce the quality of the HSI and increase the difficulty of subsequent processing, such as target detection, agriculture assessment, mineral exploitation and ground-cover classification. Therefore, noise reduction is an essential research topic in hyperspectral images analysis.

Over the past two decades, various approaches have been developed for the noise reduction of HSI, which aim to restore the original HSI from its noisy images as accurately as possible. The traditional denoising algorithms, in two-dimensional (2D) image domain, have been widely utilized to remove the noise for HSI [6,7,8,9,10], such as wavelet transform, total variation, nonlocalmeans (NLM), K-singular value decomposition (KSVD) and block matching three-dimensional filtering (BM3D). These methods denoise the HSI band by band and destroy the latent high-dimensional structure of the HSI, which result in a great loss of spectral correlations. Especially when the spectral bands are contaminated by heavy noise, these approaches cannot effectively restore the HSI at most time.

Lam et al. [4] showed that spectral domain statistics can aid in improving the quality of the restored HSI. In this regard, some techniques treat HSI as 3D data cube rather than a 2D picture, which can fully exploit the correlations in the spectral domain. In [11,12], the kernel nonnegative Tucker decomposition and the spatial–spectral wavelet shrinkage are proposed for denoising HSI. NLM techniques based on 3D cubes are applied for the HSI recovery by exploiting the high structural sparseness/redundancy [13,14]. Total variation algorithms have been proved their effectiveness in preserving edge information for denoising HSI. In [15], the cubic total variation is developed to denoise HSI, which is achieved by exploring the 2D total variation in the spatial dimension and the 1D total variation in the spectral domain. Using spectral noise differences and spatial information differences, a spectral-spatial adaptive total variation model is proposed [16]. Nonlocal models, integrated with adaptive multidimensional total variation are shown in [17]. Noise reduction based on the tensor factorization is given in [18]. The block matching 4D (BM4D) filtering is considered in [19], which reduces the noise for the volumetric data by grouping similar cubes and exploring the collaborative filtering paradigm. By solving a Bayesian least squares optimization problem, Monte Carlo sampling has been used to estimate the posterior probability for denoising HSI [20]. In [21], an algorithm employing the hybrid conditional random field model has been introduced. To improve the denoising performance, a metric *Q*-weighted fusion algorithm is proposed to merge the denoising results of both spatial and spectral views [22].

Notably, the works based on sparse dictionary learning have proven their efficacy and popularity for image recovery. The success can be attributed to the fact that valid data in corrupted images are intrinsically sparse and the noises are uniformly spread in many domains [23]. Based on this view point, the noise can be successfully reduced. The 3D sparse coding is exploited to denoise HSI [24], which could fully explore the spectral information by extracting the different patches and obtain competitive results. In [25], the dictionary learning in a Bayesian manner is applied to recover the hyperspectral cube. Exploiting the intraband structure and the interband correlation, a joint spectral–spatial distributed sparse representation is proposed for noise reduction of HSI [26]. Sparse nonnegative matrix factorization combined with spectral–spatial information is also implemented to reduce the noise of HSI [27]. Under a new linear model exploring singular value decomposition and wavelet transform, the HSI denoising is achieved via sparse regularization [28]. In the HSI, adjacent spectral bands and spatial pixels are typically highly correlated, which exhibits the low-rank characteristics of HSI [29,30,31,32]. Using low-rank matrix recovery framework, an HSI restoration technique (LRMR) is explored to simultaneously remove various noises in HSI [31]. Zhu et al. presented an HSI mixed-noise removal method [32], which achieves the promising image quality by combining low-rank constraint and total-variation-regularized. Deep learning has been widely used in the HSI analysis [33,34]. Motivated by this, a deep dictionary learning method (DDL3+FT), consisting of the hierarchical dictionary, feature denoising and fine-turning, is developed to effectively suppress the noise in HSI [34].

However, most denoising methods are globally applied to the whole HSI or utilized on specific spectral bands obtained by partitioning the spectra with a fixed value. They obviously discard the high correlations in the spectral domain. Additionally, the real-world HSI are usually contaminated by the signal-independent noise, signal-dependent noise components or the mixed noise levels, e.g., Gaussian noise, Poisson noise, dead pixel lines and stripes. The dead pixel lines and stripes only exist in some pixels/bands, and they can be regarded as sparse noises [31]. Most studies above only focus on suppressing one or two kinds of specific image noise, such as Gaussian noise with constant variance among the bands or Poisson noise, which can not exactly depict the noise characteristics of the real-world HSI.

To address the previous limitations, this paper integrates the spectral-spatial information into the hierarchical dictionary model to efficiently suppress the existing noises in HSI. The contributions of this paper can be summarized as follows:

(1) First, we present a spectral–spatial data extraction approach for HSI based on their high structural correlations in the spectral domain. Using this approach, the spectral bands, with similar and continuous features can be adaptively segmented into the same band-subset.
(2) Second, a hierarchical dictionary model is organized by the prior distributions and hyper-prior distributions to deeply illustrate the noisy HSI. It depicts the noiseless data by utilizing the dictionary, in which the spatial consistency is exploited by Gaussian process. Meanwhile, by decomposing the noise term into Gaussian noise term and sparse noise term, the proposed method can well represent the intrinsic noise properties.
(3) Last but not the least, many experiments performed under different conditions are displayed. Compared with other state-of-the-art denoising approaches, the suggested method shows superior performance on suppressing various noises, including Gaussian noise, Poisson noise, dead pixel lines and stripes.

The remainder of this paper is organized as follows: the denoising framework for 2D images based on sparse learning is presented in Section 2. The spatial-spectral data extraction and the proposed hierarchical sparse learning model, along with the Bayesian inference implemented by Gibbs sampler, are explained in detail in Section 3. Experimental results on both synthetic and real-world hyperspectral images are reported in Section 4, with comparisons between the suggested method and the state-of-the-art approaches. The concluding remarks are given in Section 5.

## 2. Sparse Learning for Denoising 2D Images

Let ***X*** represents a 2D noisy image of size *T*_1_ × *T*_2_. The common restoration algorithms regard that the pixels extracted from the image are similar to the pixels extracted from their neighboring region to some degree. In this sense, the observed images are often divided into many 2D patches before restoration. By transforming the *i*-th patch of ***X*** into the column vector ***x****_i_*, the commonly used image degradation framework can be formulated by Equation (1):

(1) $$x_{i}=Da_{i}+N$$

The first term on the right side of the equation represents the clean component of the *i*-th patch, where ***D*** is the underlying dictionary and ***a****_i_* are the sparse coefficients with respect to dictionary elements. The second part ***N*** defines the zero-mean Gaussian noise component, with the standard variance *σ*. Generally, the noiseless signals can be estimated by solving the *l_p_*-minimization problem given by Equation (2):

(2) $$\left\{D,\hat{a_{i}}\right\}=\arg\;\underset{D,a}{\min}{\left\|x_{i}-Da_{i}\right\|}_{2}^{2}+\lambda {\left\|a_{i}\right\|}_{p}$$

In Equation (2), ${\left\|x_{i}-Da_{i}\right\|}_{2}^{2}$ is the data fidelity item, which stands for the fidelity between the observed noisy image and the original clean image, and ${\left\|a_{i}\right\|}_{p}$ is the regularization item, which gives the prior knowledge of noiseless images. *λ* ≥ 0 is a regularization parameter, which is utilized to trade off between the fidelity item and sparseness of the coefficient. A larger *λ* usually implies a much sparser solution of ***a***.

Denoising approaches aim to restore the noise-free patch ${\hat{x}}_{i}$ from degraded data ***x****_i_* by calculating ${\hat{x}}_{i}=D{\hat{a}}_{i}$. The sparse prior, *p* is usually set to zero or one. When *p* = 0, ${\left\|a_{i}\right\|}_{0}$ represents the number of nonzero elements in ***a****_i_*; when *p* = 1, ${\left\|a_{i}\right\|}_{1}$ is the sum of elements’ absolute value. *l*_0_ norm is not convex, and it cannot guarantee the uniqueness of the solution. Meanwhile, *l*_0_-minimization is an NP-hard combinatorial search problem in general case, which is usually solved by greedy algorithms. *l*_1_ norm is a convex function. The uniqueness of solution can be guaranteed by replacing *l*_0_ norm with *l*_1_ norm [35]. Traditional convex optimization methods are used to solve the problem by alternating minimization of ***D*** and ***a****_i_*.

According to the types of dictionary basis, methods can be broadly classified into two groups [36]: (1) transformation-based methods. In these works, the mathematical model are developed to predict a dictionary, e.g., discrete cosine transformation (DCT), wavelet, curvelet, contourlet and shearlet; (2) learning-based methods [9,37]. The learning techniques are utilized to construct the dictionary from the training data in these studies. The learned dictionary has shown better performance in many applications. Once ***D*** and ${\hat{a}}_{i}$ are given, the idea image ***x****_i_* can be obtained as ${\hat{x}}_{i}=D{\hat{a}}_{i}$. Bayesian methods have been widely used for learning the sparse dictionary [25,38], which have achieved competing restoration performance.

All these conventional methods have achieved good performance on denoising 2D images, but for more complicated pictures, e.g., hyperspectral images, the conventional 2D methods usually have less ability to explain the structural or textural details, which significantly limits the ability of representation and the range of application for the dictionary learning. In this case, the hierarchical dictionary learning methods have been introduced to deeply exploit the latent characteristics of images [37,38,39], with promising results.

## 3. HSI Denoising with Spectral-Spatial Information and Hierarchical Sparse Learning

**Figure 1:**
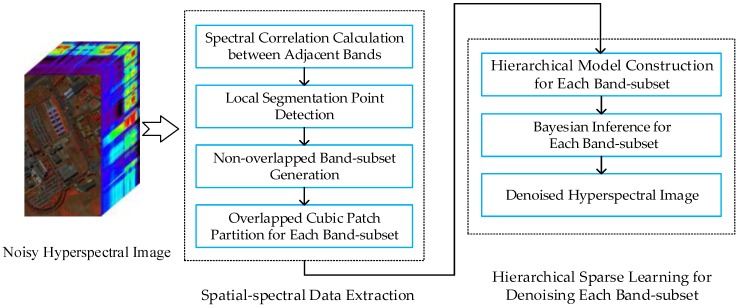
Framework of the proposed approach.

For restoring well the HSI, we extend the denoising framework for 2D images into the hierarchical dictionary learning framework in a Bayesian manner. Let ***Y*** be a HSI with a size of *l_m_* × *l_n_* × *l_λ_*, where *l_m_* × *l_n_* represents the number of spatial pixels, and *l_λ_* defines the size of the spectral domain. Generally, spectral correlations of HSI are of more importance than spatial characteristics, which have recently been reported to enhance performance in tracking, classification and target detection, etc. The proposed method consists of two main stages: spatial-spectral data extraction and noise-free estimation based on hierarchal spare learning. Firstly, according to the structure similarity index measure (SSIM), a band-subset partition is introduced to segment the HSI into multiple/one band-subsets. Each band-subset consists of continuous bands with high structure correlation. Then local homogeneous region can be extracted for each spectral pixel. Secondly, a hierarchical sparse learning model, which is composed of the clean image term, Gaussian noise term and sparse noise term, is constructed to suppress well the various noises. To effectively capture the latent spatial information of HSI, a Gaussian process with gamma constraints is imposed to the dictionary in the clean image term. The second and the third term are used to infer the statistics characteristics of existing noises, e.g., Gaussian noise, Poisson noise, dead pixel lines, stripes or a mixture of them. By solving this framework in a Bayesian manner, the restored results for each band-subset can be obtained. The framework of the proposed method is shown in Figure 1.

### 3.1. Spatial-Spectral Data Extraction

In HSI, the neighboring bands are acquired under relatively similar sensor conditions, and they are strongly correlated in the spectral domain. Based on this, SSIM is utilized to measure spectral correlations between adjacent bands [40]. Suppose ***B****_j_* and ***B****_j_*_+1_ represent 2D images lying in the *j*-th and *j*+1-th band, respectively. Structure similarity between the *j*-th and *j*+1-th band can be defined by Equation (3):

(3) $$SSIM(B_{j},B_{j+1})=\frac{\left(2\mu_{B_{j}}\mu_{B_{j+1}}+c_{1}\right)\left(2\sigma_{B_{j},B_{j+1}}+c_{2}\right)}{\left(\mu_{B_{j}}^{2}+\mu_{B_{j+1}}^{2}+c_{1}\right)\left(\sigma_{B_{j}}^{2}+\sigma_{B_{j+1}}^{2}+c_{2}\right)}$$

**Figure 2:**
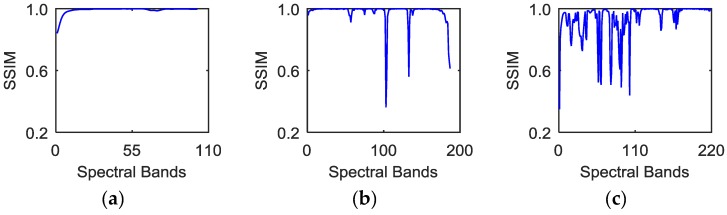
Structural correlation curves of different hyperspectral images: (**a**) Pavia data; (**b**) Urban data. (**c**) Indian Pines data.

In Equation (3), $\mu_{B_{j}}$ and $\sigma_{B_{j}}$ are the mean and variance of band ***B****_j_*, respectively; $\mu_{B_{j+1}}$ and $\sigma_{B_{j+1}}^{2}$ are the mean and variance of band ***B****_j+_*_1_; The predefined constants *c*_1_ and *c*_2_ are applied to stabilize the division with weak denominator. Normally, the closer *SSIM*(***B****_j_*,***B****_j+_*_1_) is to one, the stronger the structural correlations are between the *j*-th and *j*+1-th spectral bands. Supposing ***S****_c_*(*j*) = *SSIM*(***B****_j_*,***B****_j+_*_1_), the structural correlation curve ***S****_c_* can be generated. Figure 2 shows the structural correlation curves of different hyperspectral images.

Based on the curves in Figure 2, it can be found that the correlation coefficients across adjacent bands obviously vary considerably for different hyperspectral images. The curve in Figure 2a displays a relatively steady trend. Obvious drops exist in Figure 2b,c, which means that some adjacent spectral bands in Urban data and Indian Pines data have much lower structural similarity; meanwhile, continuous spectral bands between two neighboring drops show a relatively stable trend. However, most previous studies neglect this property of spectral bands. These approaches are applied to directly restore the entire bands, or to sequentially denoise the band-subset constructed by partitioning all spectral bands with a fixed value. To make the best of the correlations across neighboring bands, this paper explores the optimal partition in spectral domain by estimating the drops in curve ***S****_c_*. The detailed procedure of spatial-spectral data extraction is as follows: firstly, form the structural correlation curve ***S****_c_* using Equation (3). Secondly, detect the local segmentation points ***S****_c_*(*j*) in the curve ***S****_c_*. ***S****_c_*(*j*) meets the condition denoted by Equation (4):

(4) $$\left\{ \begin{array}{l} S_{c}\left(j-1\right)-S_{c}\left(j\right)>\eta \\ S_{c}\left(j+1\right)-S_{c}\left(j\right)>\eta \end{array}\right.$$

In Equation (4), *η* is a predefined threshold, which is applied to avoid the local disturbance caused by noise in curve ***S****_c_*. The beginning and ending points in ***S****_c_* can be treated as the intrinsic local partition points. To better exploit useful spectral characteristics and suppress the noise [40], a fused image is generated by using the average of the spectral bands between the neighboring segmentation points. Based on Equations (3) and (4), it is determined whether adjacent fused images are to be merged or not. By this way, the segmentation points in curve ***S****_c_* can be identified.

As shown in Figure 2a, the correlations across all neighboring bands have a relatively stable trend, and there is no need for spectral band segmentation in this case. As shown in Figure 2b,c, the HSI can be divided into the non-overlapping band-subsets based on the segmentation points. In this case, the HSI itself can be considered as a special band-subset. Let *C* denote the number of band-subsets. Above all, the noisy data can be reshaped as ***X*** = {***X***^1^,…, ***X****^c^*,…, ***X****^C^*}, *c* = 1,…, *C* and ***X****^c^* is the *c*-th band-subset.

Finally, to effectively preserve the local details of HSI in the spatial domain [41], we utilize the cubic patches instead of the 2D patches during the denoising process. By this, each band-subset is divided into many overlapped cubic patches. The size of each cubic patch is fixed as *l_x_* × *l_y_* × *l_c_*, where *l_x_* × *l_y_* is the size of spatial dimensions and *l_c_* defines the number of spectral bands in the *c*-th band-set. We note that there are various material categories within the same cubic patch, which increase the spectral-signature contamination (mixing/blurring) as the spatial size of cubic patches gets bigger. Therefore, the larger *l_x_* and *l_y_* may result in the instability of classification accuracies. If the neighborhood size is too small, it cannot well explore the spatial information. In this paper, we make a balance between the stability and the exploitation of spatial information, and we set *l_x_* = *l_y_* = 4 in the experiments.

After reshaping the cubic patches into the vectors, we can obtain the *c*-th band-subset $X^{c}=\left[x_{1}^{c},\ldots ,x_{i}^{c},\ldots ,x_{M}^{c}\right]$, where *M* = (*l_m_* − *l_x_* + 1)(*l_n_* − *l_y_* + 1) represents the number of cubic patches and $x_{i}^{c}\in R^{P},$ *P* = *l_x_* × *l_y_* × *l_c_* is the vector generated by the *i*-th cubic patch of the *c*-th band-subset.

### 3.2. Hierarchical Sparse Learning for Denoising Each Band-Subset

In this subsection, the hierarchical sparse learning framework is well constructed with prior distribution and hyper-prior distribution to restore the noisy HSI [27,37,42], which integrates the Gaussian process and Gamma distribution into dictionary atoms to explore the spatial consistency of HSI simultaneously. The novel prior consisting of prior distribution and hyper-prior distribution is often called as hierarchical prior. Noticed that, the sparse learning framework with multiple hierarchical priors can be regarded as a special case of deep learning. Considering the dataset ***X*** = {***X***^1^,…, ***X****^c^*,…, ***X****^C^*}, *c* = 1,…, *C*, the suggested approach sequentially depicts the characteristics of each subset to recover HSI by utilizing independent draws. For the *c*-th band-subset $X^{c}=\left[x_{1}^{c},\ldots ,x_{i}^{c},\ldots ,x_{M}^{c}\right]$, the hierarchical denoising model can be denoted as Equation (5):

(5) $$X^{c}=D^{c}A^{c}+N^{c}+Q^{c}\circ S^{c}$$

where the symbol ∘ represents the element-wise multiplication. The first term on the right side of the Equation (5) represents the ideally noiseless estimation for ***X****^c^*, which is represented as a linear combination of dictionary atoms. $D^{c}=\left[d_{1}^{c},\ldots ,d_{i}^{c},\ldots ,d_{K}^{c}\right]\in R^{P\times K}$ has columns that denote the dictionary atoms to be learned, where *K* is the number of dictionary atoms. In the context of HSI, the signatures of $x_{i}^{c}$ have the highly spatial consistency with samples located at its neighboring region. To better learn the HSI [43], this prior knowledge is explicitly imposed to the dictionary atom $d_{k}^{c}$ by using the Gaussian process (GP), which is constrained by the Gamma distribution. Additionally, there is a very high probability that the spectral pixels with similar characteristics in a local region share the same dictionary atoms, which is consistent with the cognition of vision and structure. $A^{c}=\left[a_{1}^{c},\ldots ,a_{i}^{c},\ldots ,a_{M}^{c}\right]\in R^{K\times M}$ places a sparse restriction on the vectors to remove noise, which can be written as ***A****^c^* = ***W****^c^*∘***Z****^c^*. The matrix $W^{c}=\left[w_{1}^{c},\ldots ,w_{i}^{c},\ldots ,w_{M}^{c}\right]$, with the size of *K* × *M*, represents the weight of matrix ***A****^c^*, where $w_{i}^{c}$ is drawn from Gaussian distribution. The sparseness of matrix ***A****^c^* is adjusted by $Z^{c}=\left[z_{1}^{c},\ldots ,z_{i}^{c},\ldots ,z_{M}^{c}\right]\in {\left\{0,1\right\}}^{K\times M}$, which is drawn from the Beta-Bernoulli process. Let symbol ∼ define an i.i.d. draw from a distribution, *N* represents the Normal distribution, ***I****_K_* is the *K* × *K* identity matrix, Bern and Beta refer to the Bernoulli and Beta distributions, respectively. Then the noise-free data ***D****^c^**A**^c^* may be represented in the following manner:

(6) $$\begin{array}{l} d_{k}^{c}∼N\left(0,\Sigma \right)\;\Sigma_{jj\prime }=\xi_{1}\exp(-{\left\|x_{j}^{c}-x_{j\prime }^{c}\right\|}_{2}^{2}/\xi_{2})\;\xi_{1}∼\Gamma \left(\beta ,\rho \right)\\ w_{i}^{c}∼N\left(0,I_{K}/\gamma_{w}^{c}\right)\;z_{ki}^{c}∼Bern\left(\pi_{k}^{c}\right)\;\pi_{k}^{c}∼Beta\left(a_{0}/K,b_{0}\left(K-1\right)/K\right) \end{array}$$

where *ξ*_1_ is drawn from the Gamma distribution and *ξ*_1_ is the predefined constant. Both of them are reapplied to manifest the smoothness between $x_{j}^{c}$ and $x_{j\prime }^{c}$. If ${\left\|x_{j}^{c}-x_{j\prime }^{c}\right\|}_{2}^{2}$ is small, and the corresponding components between $x_{j}^{c}$ and $x_{j\prime }^{c}$ are regarded to have a great spatial consistency. According to this, both the spectral correlation and spatial consistency for the *c*-th band-subset ***X****^c^* can be exploited simultaneously. $z_{ki}^{c}$ denotes whether the atom $d_{k}^{c}$ is utilized to represent $x_{i}^{c}$ with probability $\pi_{k}^{c}$ or not. When *K*→∞, the expectation *E*($\pi_{k}^{c}$) = *K*^−^^1^*a*_0_/[*K*^−^^1^*a*_0_ + *K*^−^^1^(*K*−1)*b*_0_] is approximately zero. According to this, most elements in the set ${\left\{z_{ki}^{c}\right\}}_{k=1,\ldots ,K}$ are equal to zero, and the sparsity is reasonably imposed to the vector $a_{i}^{c}$. Obviously, $a_{i}^{c}$ consists of a few non-zero values and numerous zero values. In this sense, the constraint constructed by the Beta-Bernoulli process can be regarded as a special type of *l*_0_ norm. Notice that, noiseless estimation for each $x_{j}^{c}$ uses a specific subset of dictionary atoms ${\left\{d_{k}^{c}\right\}}_{k=1,\ldots ,K}$, which is specified by the sparse vector ${\left\{z_{ki}^{c}\right\}}_{k=1,\ldots ,K}$. The expression $\left\{z_{ki}^{c}\right\}=1$ indicates that the atom $d_{k}^{c}$ is employed for representing coefficient $a_{ki}^{c}$. By analyzing the number and the locations non-zero elements in the set ${\left\{z_{ki}^{c}\right\}}_{k=1,\ldots ,K}$, the size of the dictionary can be learned adaptively, including atom selection and prediction. The matrix $N^{c}=\left[n_{1}^{c},\ldots ,n_{i}^{c},\ldots ,n_{M}^{c}\right]$, with the size of *P* × *M,* defines the zero-mean Gaussian noise. $n_{i}^{c}$ is drawn from the zero-mean Gaussian noise components, with standard variance *γ_n_*, and it can be displayed by the expressions in Equation (7). Additionally, Poisson noise is a dependent-signal noise, which means the pixels with higher light can be very strongly interrupted. To remove the dependency of the noise variance, the variance stability transform (VST) is introduced to convert Poisson noise into Gaussian noise before implementing a denoising approach [14,44]. After recovery, a corresponding inverse transformation is utilized to obtain the final HSI restoration results.

(7) $$n_{i}^{c}∼N\left(0,I_{P}/\gamma_{n}^{c}\right)$$

The third term denotes the sparse noise, such as dead pixel lines, which exploits the Beta-Bernoulli process to manifest the sparseness as displayed in Equation (8). The matrix $Q^{c}=\left[q_{1}^{c},\ldots ,q_{i}^{c},\ldots ,q_{M}^{c}\right]$, with the size of P×M, represents the intensity of the sparse noise, and $S^{c}=\left[s_{1}^{c},\ldots ,s_{i}^{c},\ldots ,s_{M}^{c}\right]\in R^{P\times M}$ depicts the location information where the sparse noise may exist.

(8) $$q_{i}^{c}∼N\left(0,I_{P}/\gamma_{v}^{c}\right)\;s_{pi}^{c}∼Bernoulli\left(\theta_{pi}^{c}\right)\;\theta_{pi}^{c}∼Beta\left(a_{\theta },b_{\theta }\right)$$

When $s_{pi}^{c}=1$, the *m*-th element of $x_{i}^{c}$ is polluted by sparse noise with the amplitude $q_{pi}^{c}$. The expectation *E*($\theta_{i}^{c}$) = *a_θ_*/(*a_θ_* + *b_θ_*) could be close to zero by adjusting the shape parameters of the Beta distribution (*a*_0_ and *b*_0_). Each element of $s_{i}^{c}$ is i.i.d. drawn from the Beta distribution separately, which depicts well the arbitrariness of positions in the sparse noise.

$\gamma_{w}^{c}$, $\gamma_{n}^{c}$ and $\gamma_{v}^{c}$ are interpretable as non-informative hyper-parameters, which can regulate the precision of $w_{i}^{c}$, $n_{i}^{c}$ and $q_{i}^{c}$. To flexibly solve the model with the posterior PDF, Gamma distribution is developed for these hyper-parameters as shown in Equation (9):

(9) $$\gamma_{w}^{c}∼\Gamma \left(c,d\right)\;\gamma_{n}^{c}∼\Gamma \left(e,f\right)\;\gamma_{v}^{c}∼\Gamma \left(g,h\right)$$

The negative logarithm posterior density function of the above model (utilized jointly to all data $X^{c}={\left\{x_{i}^{c}\right\}}_{i=1,\ldots ,M}$) may be represented as Equation (10):

(10) $$\begin{array}{l} -\log p\left(\left\{D^{c},\left\{w_{i}^{c}\right\},\left\{z_{i}^{c}\right\},\left\{q_{i}^{c}\right\},\left\{s_{i}^{c}\right\},\left\{\pi_{k}^{c}\right\},\left\{\theta_{p}^{c}\right\},\xi_{1}^{c},\gamma_{w}^{c},\gamma_{n}^{c},\gamma_{v}^{c}\right\}|\left\{x_{i}^{c}\right\}\right)=\\ \;\frac{\gamma_{n}}{2}{\sum_{i}\left\|x_{i}^{c}-D^{c}(w_{i}^{c}\circ z_{i}^{c})-q_{i}^{c}\circ s_{i}^{c}\right\|}_{2}^{2}+\frac{1}{2}{\sum_{k}\left(d_{k}^{c}\right)}^{T}\Sigma^{-1}d_{k}^{c}\\ \;+\frac{\gamma_{w}}{2}\sum_{i,k}{\left(w_{ik}^{c}\right)}^{2}+\sum_{k}Beta(\pi_{k}^{c}|a_{0}/K,b_{0}\left(K-1\right)/K)+\sum_{i,k}\log Bernoulli\left(z_{ik}^{c}|\pi_{k}^{c}\right)\\ \;+\frac{\gamma_{v}}{2}\sum_{p,i}{\left(q_{pi}^{c}\right)}^{2}+\sum_{p}Beta(\theta_{p}^{c}|a_{\theta },b_{\theta })+\sum_{p,i}\log Bernoulli\left(s_{pi}^{c}|\theta_{pi}^{c}\right)\\ \;+\log Gamma(\xi_{1}^{c}|\beta ,\rho )+\log Gamma(\gamma_{w}^{c}|c,d)\\ \;+\log Gamma(\gamma_{n}^{c}|e,f)+\log Gamma(\gamma_{v}^{c}|g,h)+const \end{array}$$

**Figure 3:**
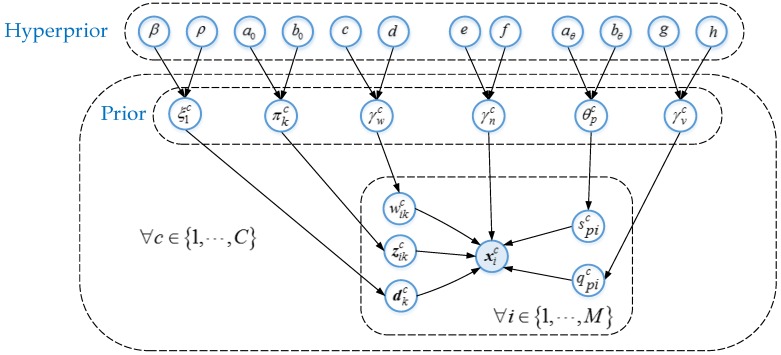
Graphical representation of the hierarchical sparse learning model.

According to Equation (10), the full posterior of all parameters can be obtained rather than a point approximation, which is commonly applied by maximum a posteriori (MAP). All necessary parameters for solving the model can be learned by the prior and hyper-prior distribution, so the proposed hierarchical sparse learning framework is more robust and accurate. A graphical representation of the complete model is shown in Figure 3.

Gibbs sampling is then implemented to solve the hierarchical model by employing the conjugacy of the model posterior distributions [45,46]. The update equations for each random variable are drawn from the conditional distributions on the most recent values of all other ones in the model. The details of the update equations can be found in the Appendix. After performing the Gibbs sampling, ***D****^c^* and ***A****^c^* can be obtained based on $\left\{d_{k}^{c}\right\}$, $\left\{w_{i}^{c}\right\}$ and $\left\{z_{i}^{c}\right\}$. Then restored images for the *c*-th band-subset $X^{c}=\left[x_{1}^{c},\ldots ,x_{i}^{c},\ldots ,x_{M}^{c}\right]$ can be inferred by calculating ***D****^c^**A**^c^*. Additionally, as there are several solutions for the same spectral pixel due to the usage of overlapping cubic patches, the final restored result is constructed by averaging all overlapping cubic patches. For all band-subsets ***X*** = {***X***^1^,…, ***X****^c^*,…, ***X****^C^*}, *c* = 1,…, *C*, the whole HSI after denoising can be obtained by sequentially performing this operation.

## 4. Experimental Results and Discussion

To evaluate the performance of the proposed approach, six state-of-the-art denoising methods were selected for a comparison based on both simulated and real data. The compared methods include K-SVD [9], BM3D [10], ANLM3D [13], BM4D [19], LRMR [31] and DDL3+FT [34]. The necessary parameters in the K-SVD, BM3D, ANLM3D and DDL3+FT methods, were finely tuned or selected automatically to generate the optimal experimental results as the reference suggested. In BM4D, the noise variance is chosen from the set {0.01, 0.03, 0.04, 0.05, 0.07, 0.09, 1.1}. In LRMR, the rank of the noiseless matrix is selected from {4, 5, 6, 7}, and the cardinality of the sparse term is chosen from the set {0, 500, 1000, 1500, 2000, 3000, 4000, 5000}. For the proposed method, *K* is set to a relatively small value to reduce computational time, and here we choose *K* = 128. The parameters of the hyper-priors in the Gaussian process are set to *β* = *ρ* = 10^−^^6^ and *ξ*_2_ = 200. The iteration number of Gibbs sampling is set to 100. For setting the remaining parameters of the hyper-priors, two cases are taken into consideration: (1) The HSI is contaminated by Gaussian noise, dead pixel lines or a mixture of them. In this case the parameters are set as follows: *a*_0_ = *b*_0_ = *c* = *d* = 10^−^^6^; *e* = *f* = 10^−^^6^; *a_θ_* = *b_θ_* = *g* = *h* = 10^−^^6^; (2) The HSI is contaminated by a mixture of Gaussian and Poisson noise or a mixture of Gaussian noise, Poisson noise and dead pixel lines. The parameters are set as follows: *a*_0_ = *b*_0_ = 10^−^^4^; *c* = d = 10^−^^5^; *e* = *f* = 10^−^^6^; *a_θ_* = *b_θ_* = 10^−^^4^; *g* = *h* = 10^−^^5^. Once the types of noises are selected, the parameters of the hyper-priors are then set as described above and have no need to be tuned. By using these hyper parameters, the proposed framework can efficiently learn the latent information and suppress the various noises for the input HSI by sampling the infinite prior space. For one Gibbs sampling iteration, the computational complexity of the proposed method is approximate to *O*(*K*(*P* + *M*) + *PM*). It should be pointed out that the proposed framework consumes much higher time than the six compared algorithms.

### 4.1. Experiment on Simulated Data

An image of Pavia University, acquired by the reflective optics system imaging spectrometer (ROSIS) over Pavia, northern Italy, is used for our experiments on simulated data. It consists of 103 spectral bands with a size of 610 × 340 for each band andcontains theentire spectral resolution reflectance characteristics collected from 430 nm to 860 nm in 10 nm steps. Before the simulated experiments, the gray values of the Pavia University data are scaled to the interval [0, 1].

For the sake of comparison, two kinds of evaluations are presented: (1) The visual appearances are shown to qualitatively estimate the experimental results before and after denoising, including spatial images at selected bands and spectral signatures of a few pixels; (2) The two commonly used metrics, peak signal to noise ratio (PSNR) and feature similarity index measure (FSIM) are provided to make a quantitative assessment on the denoising results. PSNR measures gray-level similarity between the restored image and reference image according to MSE. FSIM is designed to estimate the simulated result by integrating the gradient magnitude feature with the phase congruency feature from the view of human’s perceptual consistency [17,47]. Better denoising performance is indicated by higher PSNR and FSIM values.

**Figure 4:**
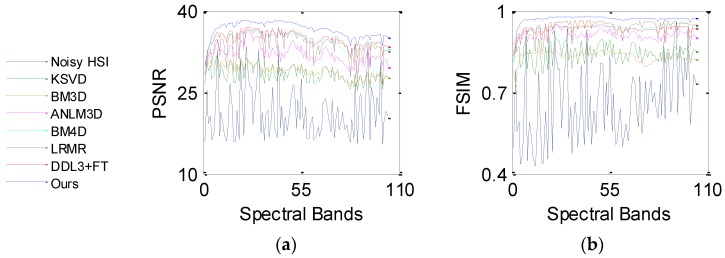
Quantitative evaluation results for Pavia data: (**a**) PSNR; (**b**) FSIM.

To estimate the effectiveness of the proposed approach, three types of noise are considered for the Pavia data: (1) zero-mean Gaussian noise; (2) Poisson noise. It is parameterized by the expression ***X****_Poisson_* = ***X*** × *peak*, where ***X****_Poisson_* refers to the noisy HSI corrupted by Poisson noise; ***X*** is the reference image; *peak* denotes the Poisson noise intensity; (3) Dead pixel lines. They are added to the same position of the selected bands in HSI, and their width varies from one line to three lines. In the simulated experiments, the noises are added for the Pavia data as the following three cases:

*Case 1*: The noise standard variance *σ* for each band of the HSI is randomly selected from 0.02 to 0.15. Figure 4 displays the PSNR and FSIM values of each band before and after recovery, which is as the quantitative evaluation for Pavia data. In Figure 4, the noisy HSI curves show big fluctuations with the spectral bands, which is caused by the changing *σ* with the spectral bands. Therefore, the noise information is very important to substantially improve the denoising performance. By adaptively predicting the noise and fully exploring the spectral-spatial information, the proposed method obtains the higher values of PSNR and FSIM than its competitors in most bands. KSVD and BM3D restore the HSI with the predefined fixed noise variance and do not learn *σ* in the simulated process. Meanwhile, they are implemented in the noisy HSI band by band and ignore the strongly spectral correlations. KSVD and BM3D show the lower values in both Figure 4a,b. ANLM3D and BM4D suppress the noise by exploiting both spatial and spectral information, which yields better simulated results compared with KSVD and BM3D. ANLM3D presents quite unstable performance, as shown in Figure 3. LRMR takes advantage of the low-rank property in HSI, and it presents a better FSIM value by retaining well the latent features of HSI. By exploring the hierarchical deep learning and fine-tuning, DDL3+FT shows comparable PSNR values as shown in Figure 4a. LRMR and DDL3+FT transform the HSI into a 2D matrix during the recovery process, and both of them cannot effectively exploit the spatial consistencies of HSI. Obviously, the plots obtained by the suggested approach have a more stable trend than the six compared approaches. This demonstrates the effectiveness and robustness of the proposed method on reducing zero-mean Gaussian noise with the *σ* varying across bands.

**Figure 5:**
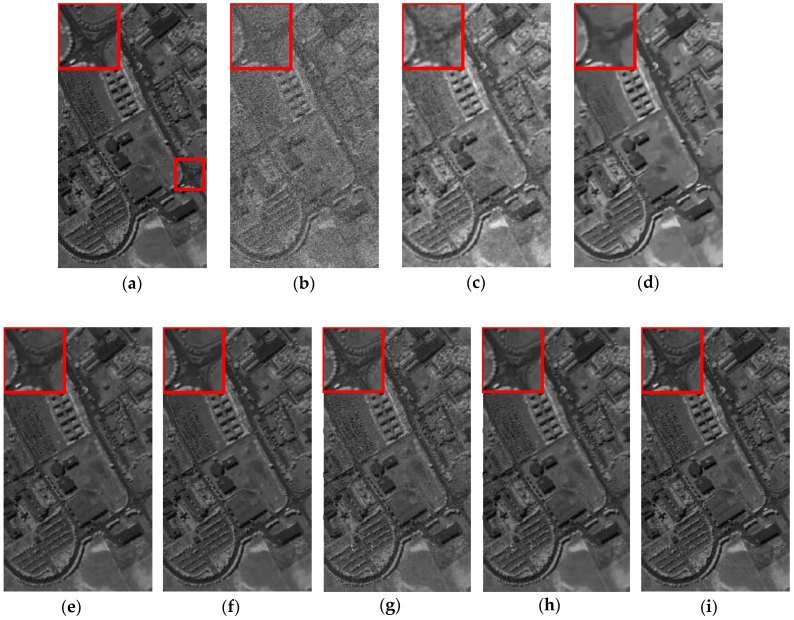
Restored images of band 101corruptedwith Gaussian noise: (**a**) clean HSI; (**b**) noisy HSI; (**c**) KSVD; (**d**) BM3D; (**e**) ANLM3D; (**f**) BM4D; (**g**) LRMR; (**h**) DDL3+FT; (**i**) Our method.

In terms of visual comparison, Figure 5 shows the denoised results of band 101 calculated by different approaches. It can be clearly seen that the proposed method works well in suppressing the noise and preserving the detailed local structure.

This is further depicted by the amplified area in the restored images of all competing methods. KSVD shows very poor performance and loses useful structural information. BM3D smooths out some important objects, which also works worse on the recovery. ANLM3D can effectively utilize the high nonlocal self-similarity to balance between smoothing and structural preservation, but it still fails to recover the profiles of local targets. The denoised result obtained by BM4D and DDL3+FT lose some fine objects. LRMR can obtain comparable results to the proposed method, but the Gaussian noise is not well reduced as shown in Figure 5g. Clearly, these visual assessment results are totally consistent with the above numerical evaluations.

**Figure 6:**
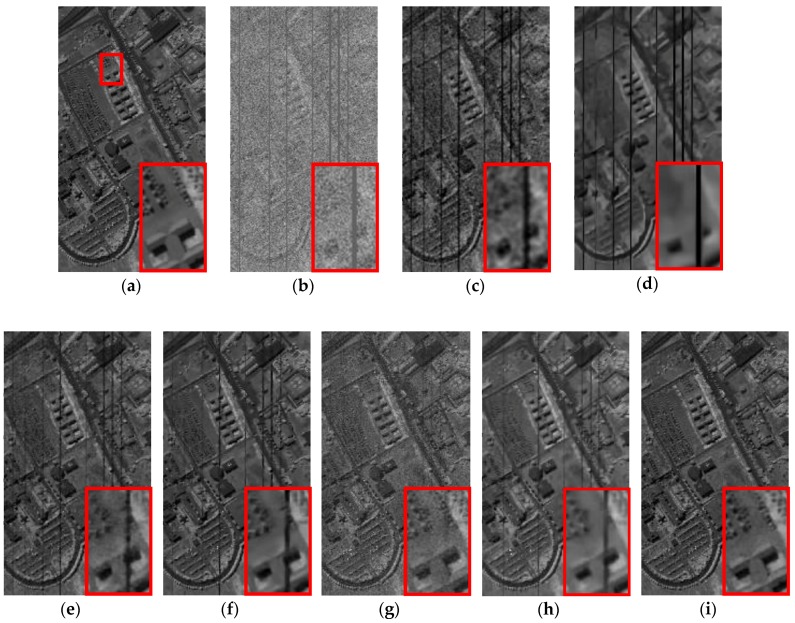
Restored results of band 95 corrupted with a mixture of Gaussian noise and dead pixel lines: (**a**) clean HSI; (**b**) noisy HSI; (**c**) KSVD; (**d**) BM3D; (**e**) ANLM3D; (**f**) BM4D; (**g**) LRMR; (**h**) DDL3+FT; (**i**) Our method.

*Case 2*: Mixed noise consisting of zero-mean Gaussian noise with standard variance *σ* = 0.2 and dead pixel lines. For Case 2, the restored results at band 95 calculated by different methods, including KSVD, BM3D, ANLM3D, BM4D, LRMR, DDL3+FT and the proposed approach, are shown in Figure 6. One area of interest is amplified in the recovery images of all methods. As shown in Figure 6a–h, we can find that the result obtained by the suggested method is much closer to the clean HSI in visual performance. The dead pixel lines in Figure 6c–h are still obvious, which means that the six compared methods fail to suppress the dead pixel lines. What’s more, according to the amplified area it can be easily observed that our restoration method can effectively restore the homogenous region while preserving the edges. KSVD can partly ameliorate the noisy image quality, but it destroys the structure of sparse coefficients in dictionary learning process, which results in the loss of edges and other structural details as shown in Figure 6c. BM3D reduces the noises by utilizing the statistics of the similar neighboring patches and achieves a much better visual impression than KSVD, but it smooths out the structural information, and its result is blurred.

ANLM3D can effectively utilize the high nonlocal self-similarity to achieve better balance smoothing and structural preserving. It fails however to preserve the edges and the details. BM4D can achieve visual improvements by utilizing the 3D nonlocal self-similarity of data cube, however, it removes some of the fine objects and over smooths the HSI. As shown in Figure 6g, the blurred black lines mean that LRMR only removes part of the dead pixels lines; meanwhile, the blurry white dots indicate that LRMR cannot efficiently remove the heavy Gaussian noise. Clearly, the DDL3+FT method fails to restore the mixed noisy image, which can be seen from the blurry edges and obvious dead pixel lines as shown in Figure 6h.

**Figure 7:**
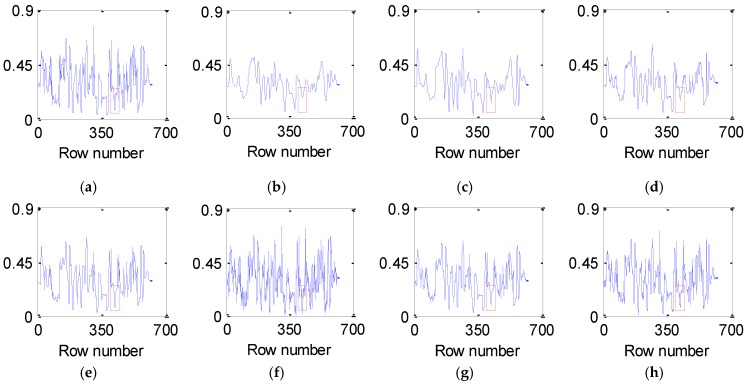
Vertical profiles of band 95 at pixel (559, 150) before and after denoising: (**a**) clean HSI; (**b**) KSVD; (**c**) BM3D; (**d**) ANLM3D; (**e**) BM4D; (**f**) LRMR; (**g**) DDL3+FT; (**h**) Our method.

**Figure 8:**
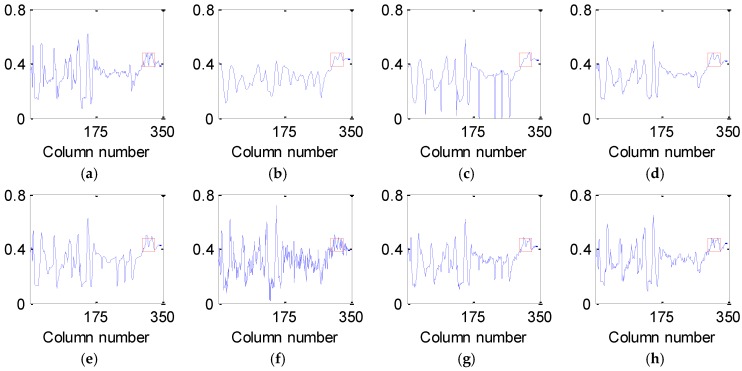
Horizontal profiles of band 95 at pixel (559, 150) before and after denoising: (**a**) clean HSI; (**b**) KSVD; (**c**) BM3D; (**d**) ANLM3D; (**e**) BM4D; (**f**) LRMR; (**g**) DDL3+FT; (**h**) Our method.

Figure 7 and Figure 8 present the vertical and horizontal profiles of band 95 at pixel (559, 150) in the simulated experiment of Case 2, respectively. Clearly, the results are visually different in both shape and amplitude, where rapid fluctuations are introduced by the existence of dead pixel lines.

From Figure 7 and Figure 8, it can be observed that the profiles produced by the proposed method are closest to those of the original HSI and give the best removal of Gaussian noise and dead pixel lines. The curves in Figure 7b–g and Figure 8b–g are not ideally consistent with those in Figure 7a and Figure 8a and therefore lead to the limited ability for denoising HSI, which greatly supports the analysis above.

**Figure 9:**
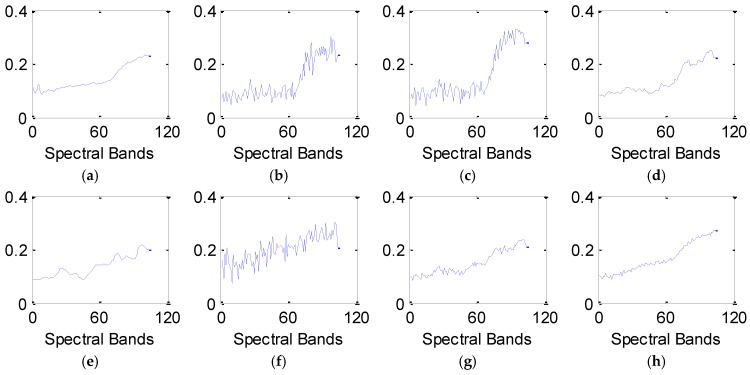
Spectral reflectance curves at pixel (559, 150) before and after denoising: (**a**) clean HSI; (**b**) KSVD; (**c**) BM3D; (**d**) ANLM3D; (**e**) BM4D; (**f**) LRMR; (**g**) DDL3+FT; (**h**) Our method.

Spectral signatures of the clean image and the restored images at pixel (559, 150) are displayed in Figure 9, which are very critical for the classification and identification of the HSI. KSVD and BM3D denoise the HSI band by band and destroy the spectral–spatial correlation. As shown in Figure 9b,c, there are recognizable artifacts in the restored signatures obtained by KSVD and BM3D. Using ANLM3D and BM4D, the restored signatures are much nearer to the initial spectral reflectance curve due to the exploitation of the spectral–spatial information, but they appear to fluctuate strongly compared with the clean spectral signature. The restored signatures LRMR and DDL3+FT have the similar trends and shapes, but the detailed information cannot be well remained. Obviously, spectral signature calculated by the proposed method is the optimal, which demonstrates the superiority of the proposed method in reducing the Gaussian noise and dead pixel lines.

**Figure 10:**
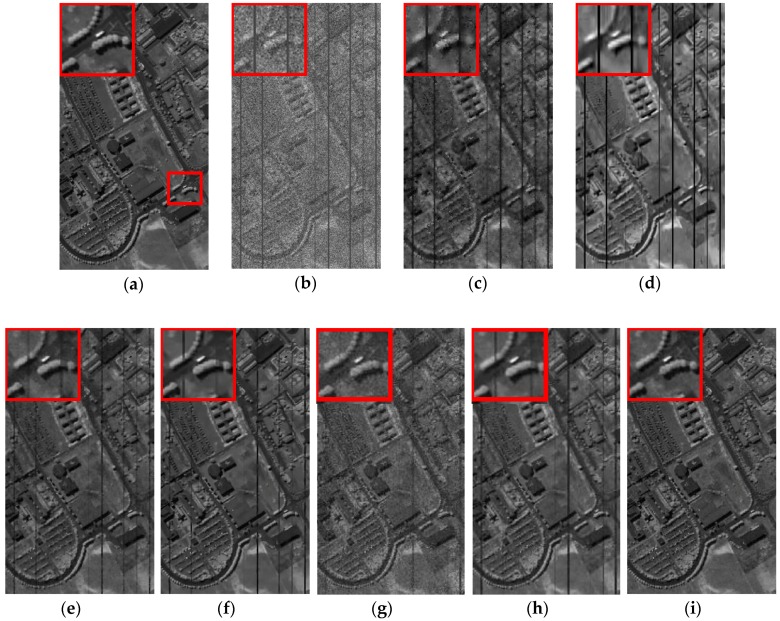
Restored results of band 90 corrupted with a mixture of Gaussian noise, Poisson noise and dead pixel lines:(**a**) clean HSI; (**b**) noisy HSI; (**c**) KSVD; (**d**) BM3D; (**e**) ANLM3D; (**f**) BM4D; (**g**) LRMR; (**h**) DDL3+FT; (**i**) Our method.

*Case 3*: Mixed noise consisting of zero-mean Gaussian noise, Poisson noise and dead pixel lines, with *σ* = 0.15 and *peak* = 30. For Case 3, band 90 of the initial HSI and denoised results are presented in Figure 10. One area of all listed images is magnified for making a clear comparison. KSVD shows poor performance as shown in Figure 10c, and BM3D is oversmoothing and loses some useful objects. Also, there are obvious dead pixel lines in Figure 10c,d, which indicates that the KSVD and BM3D methods fail to remove the dead pixel lines. The ANLM3D and BM4D algorithms can only reduce part of the dead pixel lines, as presented in Figure 10d,f. Both of them do not work well in preserving the fine objects. The restored image of LRMR still has dead pixels lines and some Gaussian noise. From Figure 10h, it can be found that DDL3+FT fails in suppressing the dead pixels lines. As presented in Figure 10i, our approach can effectively remove the Gaussian noise, Poisson noise and dead pixel lines while preserving the local details such as edges and textures. Obviously, it performs the best compared to the six compared methods.

**Figure 11:**
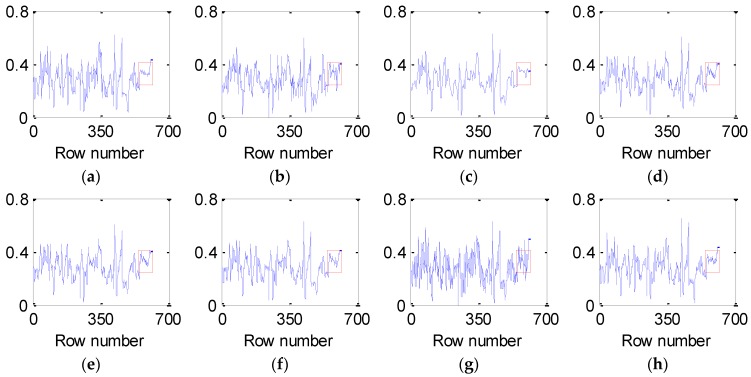
Vertical profiles of band 90 at pixel (399, 290) before and after denoising: (**a**) clean HSI; (**b**) KSVD; (**c**) BM3D; (**d**) ANLM3D; (**e**) BM4D; (**f**) LRMR; (**g**) DDL3+FT; (**h**) Our method.

**Figure 12:**
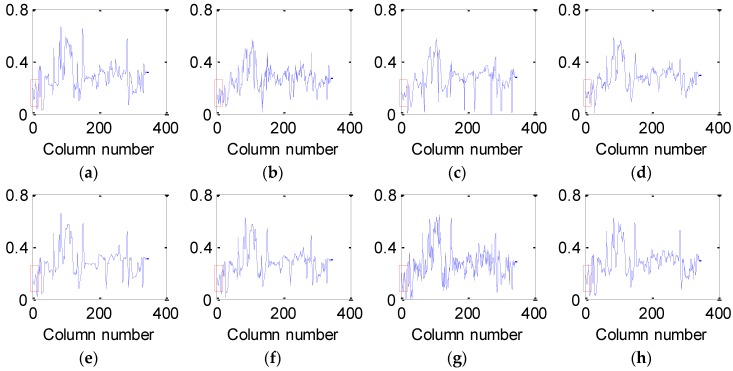
Horizontal profiles of band 90 at pixel (399, 290) before and after denoising:(**a**) clean HSI; (**b**) KSVD; (**c**) BM3D; (**d**) ANLM3D; (**e**) BM4D; (**f**) LRMR; (**g**) DDL3+FT; (**h**) Our method.

**Figure 13:**
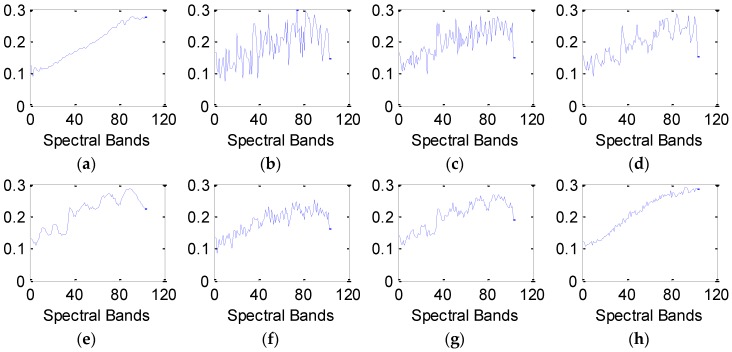
Spectral reflectance curves before and after denoising at pixel (399, 290): (**a**) clean HSI; (**b**) KSVD; (**c**) BM3D; (**d**) ANLM3D; (**e**) BM4D; (**f**) LRMR; (**g**) DDL3+FT; (**h**) Our method.

Figure 11 and Figure 12 display the vertical and horizontal profiles of band 90 at pixel (399, 290) in simulated experiment of Case 3, separately. The spectral reflectance curves of all competing approaches at location (399, 290) before and after denoising are shown in Figure 13.

The rapid fluctuations of results in Figure 10 are caused by the dead pixel lines. A visual comparison is made based on the difference in the shape and amplitude of Figure 11, Figure 12 and Figure 13. KSVD is inferior to suppress the mixed noise and preserve the spectral information, which can be clearly seen in Figure 11b, Figure 12b and Figure 13b. According to Figure 11c, Figure 12c and Figure 13c, BM3D is over smoothing while preserving the structure and introduces some recovery artifacts. From Figure 11d, Figure 12d and Figure 13d, it can be found that ANLM3D can partly reduce the mixed noise, which can be easily seen by the reduction of rapid fluctuations as shown in Figure 12d. As shown in Figure 11e,f Figure 12e,f and Figure 13e,f, the curves obtained by BM4D and LRMR are much near to the initial curves compared to KSVD, BM3D, ANLM3D and DDL3+FT, but they fail in the detail preservation. By comparing the region marked by red rectangle, it is easily obtained that our method achieves the best approximation to the intrinsic patterns of the clean HSI, which is fully consistent with the analysis above.

### 4.2. Experiment on Real Data

Two well-known real data sets are adopted for evaluating the proposed method, including Urban data and Indian Pines data. The visual impressions are presented to qualitatively estimate the experimental results before and after restoration. For real HSI, there is no reference image to implement the numerical evaluation, e.g., PSNR and FSIM. Hence, the classification accuracies on Indian Pines data are utilized to quantitatively estimate the denoising performance.

#### 4.2.1. Denoising for Urban Data

Urban data, with the original size of 307 × 307 × 210, is acquired by the HYDICE sensor. Due to the detector-to-detector difference, it has different intensity strips and mixed noises versus bands. After removing the bands 104–108, 139–151, and 207–210 of Urban data, polluted by the atmosphere and water absorption, a subset with the size of 150 × 150 × 188 is used in the following experiments.

**Figure 14:**
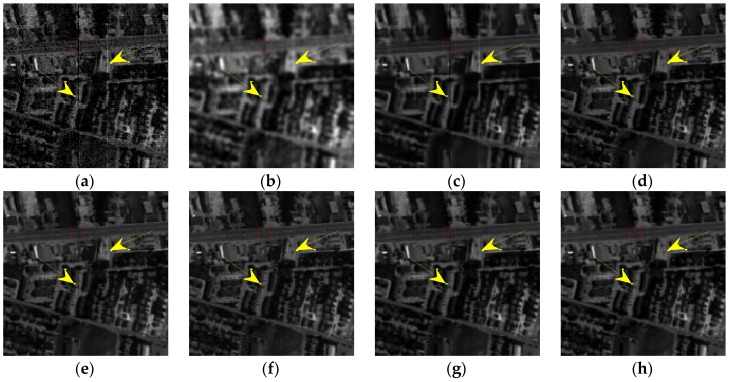
Restored results in Urban image: (**a**) Original band 186; (**b**) KSVD; (**c**) BM3D; (**d**) ANLM3D; (**e**) BM4D; (**f**) LRMR; (**g**) DDL3+FT; (**h**) Our method.

**Figure 15:**
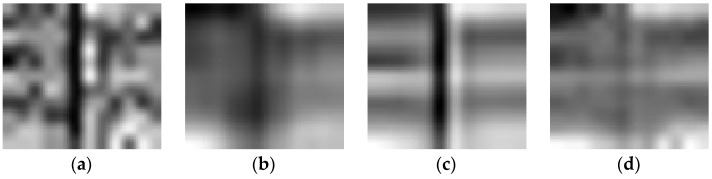

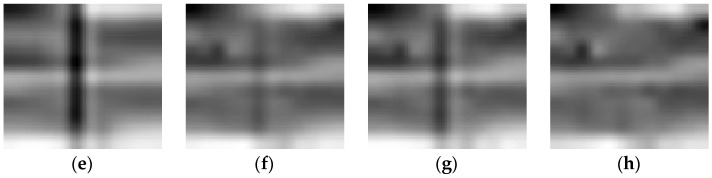
×4 magnified results of the various approaches in the red rectangle of Figure 14: (**a**) Original band 186; (**b**) KSVD; (**c**) BM3D; (**d**) ANLM3D; (**e**) BM4D; (**f**) LRMR; (**g**) DDL3+FT; (**h**) Our method.

Figure 14 presents the recovered images of band 186 obtained by different methods. To facilitate the visual comparison, the yellow arrows are utilized to mark the obvious stripes in Figure 14. Meanwhile, Figure 15 gives the enlarged details in the red rectangle of Figure 14. KSVD shows poor performance on the reduction of stripes and the preservation of structures as shown in Figure 14b and Figure 15b. Obviously, BM3D and BM4D smooth out the important texture and fine targets, and both of them fail in removing the stripes. According to Figure 14d,g and Figure 15d,g, it can be found that ANLM3D and DDL3+FT can partly suppress the stripes, and DDL3+FT has a better ability for preserving the texture and edge information than ANLM3D. LRMR shows better recovery performance than the other five compared methods on the noise reduction and structure preservation. From Figure 14f,h, it can be clearly observed that LRMR is inferior to the proposed method in removing the stripes. Generally, our proposed restoration method can efficiently restore the Urban data and convincingly outperform the six compared denoising approaches.

**Figure 16:**
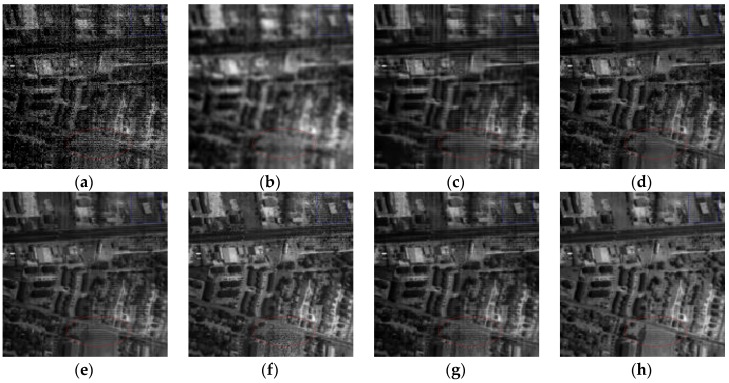
Restored results in Urban image: (**a**) Original band 104; (**b**) KSVD; (**c**) BM3D; (**d**) ANLM3D; (**e**) BM4D; (**f**) LRMR; (**g**) DDL3+FT; (**h**) Our method.

**Figure 17:**
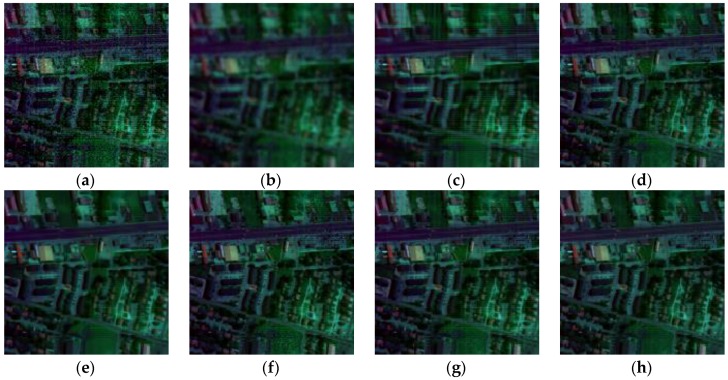
Restored results of Urban image: (**a**) original false-color image (R: 1, G: 104, and B: 135); (**b**) KSVD; (**c**) BM3D; (**d**) ANLM3D; (**e**) BM4D; (**f**) LRMR; (**g**) DDL3+FT; (**h**) Our method.

In addition, Figure 16 presents the results at band 104 before and after denoising. As shown in Figure 16a, there are many heavy stripes within the initial band 104. The false-color images of Urban data before and after restoration are presented in Figure 17, which consist of the *1*st, *104*th and *135*th bands. KSVD updates the dictionary atoms one by one, and destroys the structure of sparse coefficients. As shown in Figure 16b and Figure 17b, it blurs the image structures and shows very poor performance. By exploiting the block matching and 3D collaborative filter, BM3D greatly enhances the image quality of the initial ones, but there are still obvious stripes in Figure 16c and Figure 17c. According to the regions marked by the red ellipse, it can be found that ANLM3D, BM4D, LRMR and DDL3+FT can partly remove the stripes, and LRMR works worse in effectively restoring the images polluted by serious noises. Obviously, the proposed method shows the superior performance on denoising the flat region with the better edge preservation. Especially, by compared the regions marked by a blue rectangle in Figure 16, it can be easily found that the suggested method can restore well the targets and remain the edges while efficiently reducing the stripes and mixed noises. Above all, the restored images obtained by the proposed method present the best visual result than KSVD, BM3D, ANLM3D, BM4D, LRMR and DDL3+FT, with the satisfying detail preservation.

#### 4.2.2. Experimental Results on Indian Pines Data

The second data set is named Indian Pines, which is recorded by the NASA AVIRIS sensor over the Indian Pines region in 1992, and this dataset contains much random noise in some bands during the acquiring process. It comprises 220 spectral bands and the spatial dimension of each spectral band is 145 × 145 pixels. For Indian Pines data, the ground truth has 16 land cover classes and a total of 10,366 labeled pixels.

**Figure 18:**
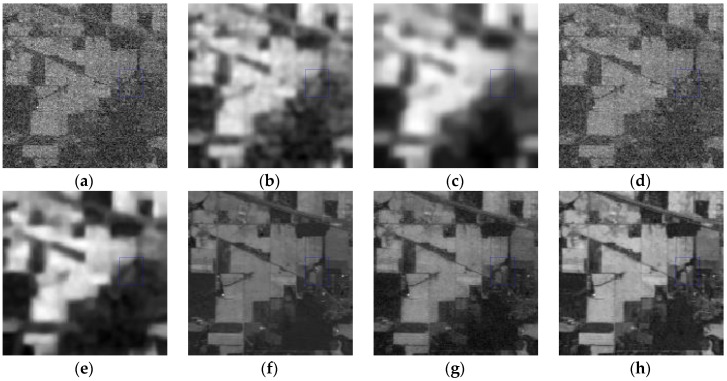
Restored results in Indian Pines image: (**a**) Original band 164; (**b**) KSVD; (**c**) BM3D; (**d**) ANLM3D; (**e**) BM4D; (**f**) LRMR; (**g**) DDL3+FT; (**h**) Our method.

**Figure 19:**
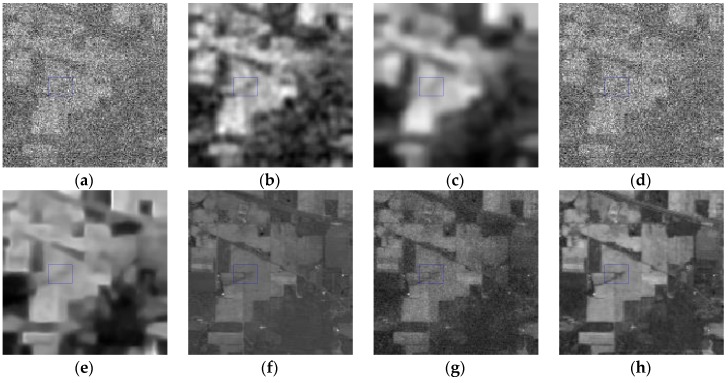
Restored results in Indian Pines image: (**a**) Original band 220; (**b**) KSVD; (**c**) BM3D; (**d**) ANLM3D; (**e**) BM4D; (**f**) LRMR; (**g**) DDL3+FT; (**h**) Our method.

**Figure 20:**
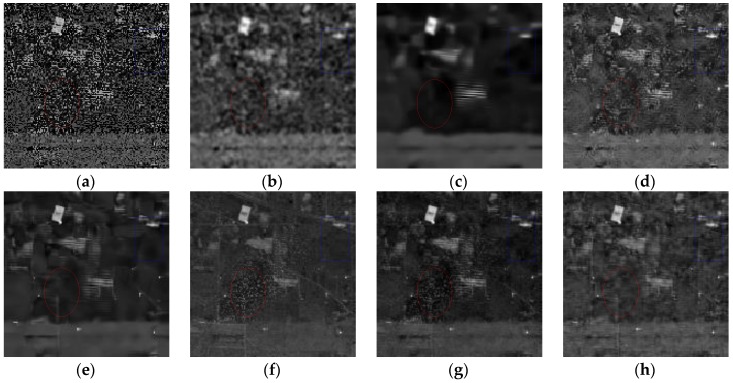
Restored results in Indian Pines image: (**a**) Original band 1; (**b**) KSVD; (**c**) BM3D; (**d**) ANLM3D; (**e**) BM4D; (**f**) LRMR; (**g**) DDL3+FT; (**h**) Our method.

Figure 18, Figure 19 and Figure 20 display the visual comparisons of different bands polluted by different noises. Obviously, the proposed method achieves better visual quality than the compared ones. From Figure 18b, Figure 19b and Figure 20b, KSVD shows poorer denoising capability than other methods. BM3D significantly blurs the images and loses the texture information and edges.

As shown in Figure 18d and Figure 19d, the image quality improvements obtained by ANLM3D appear very small and can be neglected. BM4D is oversmoothing and loses texture information. By observing the regions marked by the blue rectangle in Figure 18, Figure 19 and Figure 20, our denoising method has the better ability on the edge and structure preservation than LRMR and DDL3+FT; meanwhile, LRMR and DDL3+FT work worse in efficiently removing the random noises as shown in the regions marked by the red ellipse in Figure 18, Figure 19 and Figure 20. According to Figure 18, Figure 19 and Figure 20, our algorithm is much more superior than the six compared methods for seriously corrupted images, which is consistent with the above analysis, so our algorithm can perform best in the removal of random noises from the Indian Pines data, while effectively improving the quality of the noisy HSI and retoring the texture and structure details. For classification-based evaluation, two cases are taken into consideration according to the testing data: (1) classifying the 20 heavily corrupted bands of Indian Pines data, including bands 104–108, 150–163 and 220; (2) classifying the Indian Pines data with 20 heavily corrupted bands removed. Similar with the setting in [48], the number of training samples for small classes “alfalfa”, “grass/pasture mowed”, and “oats” is set as 15 samples per class, and the number for the remaining classes is set to 50. Support vector machine (SVM) is utilized as the classification method. As usual, the commonly used overall accuracy (OA) and the kappa coefficient are selected as evaluation metrics and the map of the results is used for visual estimation.

Table 1 lists the overall accuracy (OA) and kappa coefficient (κ) of the results for the heavily corrupted bands. Table 2 shows the OA and kappa coefficient of the results for the remaining bands. After achieving the recovery for testing data, the values of OA and kappa coefficient are obviously enhanced as shown in Table 1 and Table 2, which demonstrate the necessity of HSI denoising before implementing the classification. Compared with other algorithms, the proposed method obtains the best OA and kappa coefficients in both Table 1 and Table 2, which means that our denoising method can greatly restore the structure information (which is essential for classification) in the seriously polluted bands or the remaining 200 bands. We note that ANLM3D method obtains an OA value of 25.17% and *κ* of 0.2135 in Table 1, which are just 9.43% and 0.1223 higher than initial HSI. This lower classification accuracy and kappa coefficient are totally in line with the poor denoising performance as displayed in Figure 19d.

**Figure 21:**
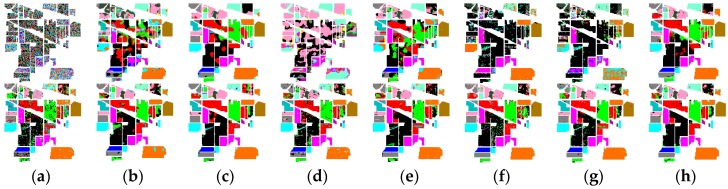
Classification results for Indian Pines data before and after denoising: the first row is the results of the 20 heavily corrupted bands; the second row presents the results of the remaining 200 bands: (**a**) Original HSI; (**b**) KSVD; (**c**) BM3D; (**d**) ANLM3D; (**e**) BM4D; (**f**) LRMR; (**g**) DDL3+FT; (**h**) Our method.

The classification maps of different algorithms are displayed in Figure 21, where the first row is the results of the 20 heavily corrupted bands and the second row presents the results of the remaining 200 bands before and after restoration. According to Figure 21, it can be easily observed that the result of suggested method presents the better visual effect than the six compared algorithms.

### 4.3. Discussion

#### 4.3.1. Threshold Parameter *η*

**Figure 22:**
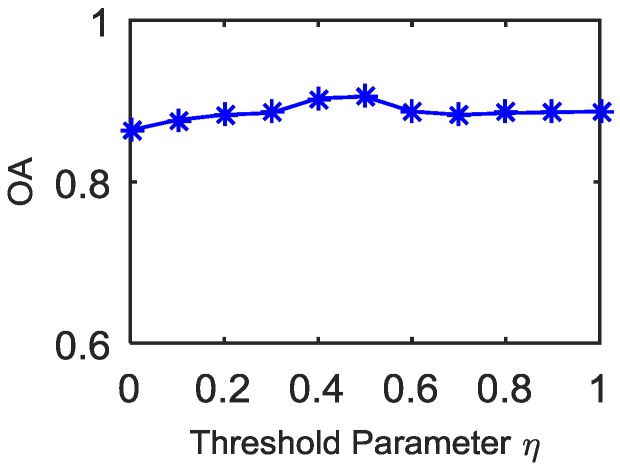
SVM classification accuracies of Indian Pines data (104–108, 150–163, 220 removed).

The value of the parameter *η* in Equation (4) is selected from 0 to 1. Quantitative evaluations of different η values were implemented by comparing the OA metric on Indian Pines data. Noticing that for the data consisting of the 20 heavily polluted bands, the SSIM values of adjacent bands are very similar with each other as shown in Figure 2c, we just analyzed the remaining 200 bands here. Figure 22 presents the relative SVM classification accuracies. It can be seen that the highest classification accuracies occur in *η* ∈ (0.4,0.5) and the lowest values is in *η* ∈ (0,0.1). In particular, the case when *η* = 0 means that the segmentation points are selected in line with the local dropping points, which result in the absence of sample information for some band-subsets and decrease the classification accuracy. When *η* = 1, there is no need to divide the bands. Observing the curve in Figure 22, it can be found that the appropriate *η* may well enhance the classification accuracies, which also demonstrates the necessary for the band-subset segmentation.

The number of the band subsets, *C*, is only dominated by the parameter *η*. For the HSI with *C* band subsets, its computational complexity is approximately equal to $O(\sum_{c=1}^{C}\left(l_{x}l_{y}l_{c}\left(K_{c}+M\right)+\left(K_{c}M\right)\right)$ for one iteration. The values of *l_x_*, *l_y_* and *M* are fixed in our experiments. Hence, the computational complexity of the proposed method is determined by the parameters *l_c_* and *K_c_*. To simplify the analysis, we consider the special case that the number of dictionary atoms, *K_c_*, is set as the same value *K’* for all band-subsets. Then the computational complexity can be rewritten as $O\left(l_{x}l_{y}\left(K^{\prime }+M\right)\left(l_{1}+\ldots +l_{C}\right)+CK^{\prime }M\right)$. It is noted that the value of the expression ($l_{1}+\ldots +l_{C})$ is the number of bands in HSI and *M* is much larger than *K*’, so the computational complexity is mainly dominated by the expression $K^{\prime }M$. Due to the strong spectral correlations, *C* is usually smaller than 10, as shown in Figure 2. When *C* changes from 1 to 10, the empirical value of *K*’ shows a rapid decrease, and the relative value of $CK^{\prime }M$ shows a downwards trend, which implies a lower computational complexity.

#### 4.3.2. The Sparse Noise Term

**Figure 23:**
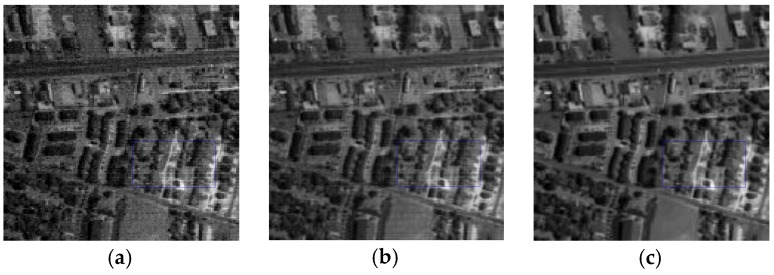
Restored results in Urban data: (**a**) Original band 133; (**b**) SNT-DIS; (**c**) Our method.

To evaluate the effectiveness of the sparse noise term, we performed a comprehensive comparison between the restored images obtained by the proposed method with the sparse noise term disabled and enabled. The Urban dataset is applied for the simulation, and the method with disabled sparse noise term is named as SNT-DIS. Figure 23 shows the visual impression of band 133 obtained by SNT-DIS and the proposed method. Compared with the corrupted image in Figure 23a, it can be easily found that both algorithms can greatly improve the image quality as displayed in Figure 23b,c, but the SNT-DIS can only reduce a small part of the stripes, as presented in Figure 23b, and it also fails in preserving the fine objects. As presented in Figure 23c, the suggested method can effectively remove heavy stripes and mixed noises while preserving the local details such as edges and textures. The obvious superiority of the proposed method demonstrates that the denoising performance can be greatly improved by introducing the sparse noise term.

## 5. Conclusions

In this paper, a novel hierarchical framework combining structural correlations in spectral domain and sparse learning is developed for HSI denoising. First, with the suggested band-subset partition, spectral-spatial cubic patches can be effectively extracted, where each patch has strong structure correlations between adjacent bands and high spatial similarities within the cubic patch. Second, noise-free estimations for each band-subset are generated by solving a sparse problem constructed by a Beta-Bernoulli process. The Gaussian process with Gamma distribution is regarded as the precursor of dictionary elements to fully exploit the spatial consistency in HSI. By utilizing the spectral-spatial information and Gibbs sampling, the suggesting framework can effectively learn the latent detail and structure information in HSI. It has the advantage of adaptively predicting the noise in a data-driven manner, which enables the framework to better depict the existing noises in HSI. Meanwhile, it can automatically infer the size and the atoms of dictionary. Compared with KSVD, BM3D, ANLM3D, BM4D, LRMR and DDL3+FT, the proposed framework achieves superior performance both numerically and visually, which has been verified on the simulated and real HSI. It can suppress various noises in HSI, such as Gaussian noise, Poisson noise, dead pixel lines, stripes or a mixture of them, with better image quality improvement and structure characteristics preservation. Further research will be directed toward exploiting the appropriately hidden structure and reducing the computational time for the analysis of the HSI.

## Figures and Tables

**Table 1:** SVM classification accuracies on heavily corrupted bands (104–108, 150–163, 220) of the Indian Pines data.

	Initial HSI	KSVD	BM3D	ANLM3D	BM4D	LRMR	DDL3+FT	Ours
OA	15.74%	57.96%	81.2%	25.17%	69.27%	48.38%	49.12%	83.76%
κ	0.0912	0.5368	0.7803	0.2135	0.679	0.3664	0.4284	0.8109

**Table 2:** SVM classification accuracies on the Indian Pines data (bands 104–108, 150–163, 220 removed).

	Initial HSI	KSVD	BM3D	ANLM3D	BM4D	LRMR	DDL3+FT	Ours
OA	74.39%	87.96%	0.9215%	81.06%	87.59%	87.18%	85.92%	90.35%
κ	0.7183	0.8531	0.8673	0.775	0.8568	0.8548	0.8442	0.8726

## Appendix A

Supposing that symbol—represents “the remaining variables except the one being sampled”. The update equations for the *c*-th band-subset are listed asfollows:

Sampling $d_{k}^{c}$:

(A1) $$p\left(d_{k}^{c}|-\right)∼N\left(d_{k}^{c}|\mu_{d_{k}^{c}},\Omega_{d_{k}^{c}}\right)$$

where the covariance $\Omega_{d_{k}^{c}}$ and mean $\mu_{d_{k}^{c}}$ are expressed as:

(A2) $$\Omega_{d_{k}^{c}}={\left(\Sigma^{-1}+\gamma_{n}^{c}\sum_{i}{\left(w_{ik}^{c}\right)}^{2}{\left(z_{ik}^{c}\right)}^{2}\right)}^{-1}$$

(A3) $$\mu_{d_{k}^{c}}=\gamma_{n}^{c}\Omega_{d_{k}^{c}}\sum_{i}w_{ik}^{c}z_{ik}^{c}x_{\left(i,-k\right)}^{c}$$

where $x_{\left(i,-k\right)}^{c}=x_{i}^{c}-D^{c}(w_{i}^{c}⊙z_{i}^{c})-q_{i}^{c}⊙s_{i}^{c}+(w_{ik}^{c}⊙z_{ik}^{c})d_{k}^{c}$.

Sampling $z_{ik}^{c}$:

(A4) $$p\left(z_{ik}^{c}|-\right)∼\mathrm{Bern}(\frac{p_{1}}{p_{1}+p_{0}})$$

The posterior probability of $z_{ik}^{c}=1$ is proportional to:

(A5) $$p_{1}=\pi_{k}^{c}\exp(-\frac{\gamma_{n}^{c}}{2}({\left(w_{ik}^{c}\right)}^{2}{\left(d_{k}^{c}\right)}^{T}d_{k}^{c}-2w_{ik}^{c}{\left(d_{k}^{c}\right)}^{T}x_{\left(i,-k\right)}^{c}))$$

The posterior probability of $z_{ik}^{c}=0$ is proportional to:

(A6) $$p_{0}=1-\pi_{k}^{c}$$

Sampling $w_{i}^{c}$:

(A7) $$p\left(w_{ik}^{c}|-\right)∼N(w_{ik}^{c}{|\mu_{wik}^{c},\Omega }_{wik}^{c})$$

(A8) $$\Omega_{wik}^{c}={\left(\gamma_{w}^{c}+\gamma_{n}^{c}{\left(z_{ik}^{c}\right)}^{2}{\left(d_{k}^{c}\right)}^{T}d_{k}^{c}\right)}^{-1}$$

(A9) $$\mu_{wik}^{c}=\gamma_{n}^{c}\Omega_{wik}^{c}{\left(d_{k}^{c}\right)}^{T}x_{\left(i,-k\right)}^{c}$$

Sampling $\pi_{k}^{c}$:

(A10) $$p\left(\pi_{k}^{c}|-\right)∼\mathrm{Beta}(a_{0}/K+\sum_{i}z_{ik}^{c},b_{0}\left(K-1\right)/K+M-\sum_{i}z_{ik}^{c})$$

Sampling $\xi_{1}^{c}$:

(A11) $$p\left(\xi_{1}^{c}|-\right)∼\Gamma (\beta +PK/2,\rho +\sum_{k}{\left(d_{k}^{c}\right)}^{T}\Sigma^{-1}d_{k}^{c}/2)$$

Sampling $\gamma_{w}^{c}$:

(A12) $$p\left(\gamma_{w}^{c}|-\right)∼\Gamma (c+MK/2,d+\sum_{i}{\left(w_{i}^{c}\right)}^{T}w_{i}^{c}/2)$$

Sampling $\gamma_{n}^{c}$:

(A13) $$p\left(\gamma_{n}^{c}|-\right)∼\Gamma (e+PM/2,f+{\sum_{i}\left\|x_{i}^{c}-D^{c}(w_{i}^{c}\circ z_{i}^{c})-q_{i}^{c}\circ s_{i}^{c}\right\|}_{2}^{2})$$

Sampling $s_{pi}^{c}$:

(A14) $$p\left(s_{pi}^{c}|-\right)∼\mathrm{Bern}(\frac{v_{1}}{v_{1}+v_{0}})$$

(A15) $$v_{1}=\theta_{p}^{c}\exp(-\frac{\gamma_{v}}{2}({\left(q_{pi}^{c}\right)}^{2}-2q_{pi}^{c}x_{\left(i,s\right)}^{c}))$$

(A16) $$v_{0}=1-\theta_{p}^{c}$$

where $x_{\left(i,s\right)}^{c}=x_{i}^{c}-D^{c}\left(w_{i}^{c}\circ z_{i}^{c}\right).$ The posterior probability that $s_{pi}^{c}=1$ is proportional to ν_1_, and the posterior probability that $s_{pi}^{c}=0$ is proportional to ν_0_.

Sampling $q_{pi}^{c}$:

(A17) $$p\left(q_{pi}^{c}|-\right)∼N(\mu_{qpi},\Omega_{qpi})$$

(A18) $$\Omega_{qpi}={\left(\gamma_{v}^{c}+\gamma_{n}^{c}{\left(s_{pi}^{c}\right)}^{2}\right)}^{-1}$$

(A19) $$\mu_{qpi}=\gamma_{n}^{c}\Omega_{qpi}s_{pi}^{c}x_{\left(i,s\right)}^{c}$$

Sampling $\theta_{p}^{c}$:

(A20) $$p\left(\theta_{p}^{c}|-\right)∼\mathrm{Beta}(a_{\theta }+\sum_{i}s_{pi}^{c},b_{\theta }+M-\sum_{i}s_{pi}^{c})$$

Sampling $\gamma_{\nu }^{c}$:

(A21) $$p\left(\gamma_{v}^{c}|-\right)∼\Gamma (g+PM/2,h+\sum_{i}{\left(q_{i}^{c}\right)}^{T}q_{i}^{c}/2)$$
